# Novel insights into chromosome evolution of Charadriiformes: extensive genomic reshuffling in the wattled jacana (*Jacana jacana*, Charadriiformes, Jacanidae)

**DOI:** 10.1590/1678-4685-GMB-2019-0236

**Published:** 2020-02-17

**Authors:** Rafael Kretschmer, Marcelo Santos de Souza, Suziane Alves Barcellos, Tiago Marafiga Degrandi, Jorge C. Pereira, Patricia C.M. O’Brien, Malcolm A. Ferguson-Smith, Ricardo José Gunski, Analía del Valle Garnero, Edivaldo Herculano Correa de Oliveira, Thales Renato Ochotorena de Freitas

**Affiliations:** 1 Universidade Federal do Rio Grande do Sul, Programa de Pós-graduação em Genética e Biologia Molecular - PPGBM, Porto Alegre, Rio Grande do Sul, RS, Brazil.; 2 University of Cambridge, Department of Veterinary Medicine, Cambridge Resource Centre for Comparative Genomics, Cambridge, United Kingdom.; 3 Universidade Federal do Pampa, Programa de Pós-graduação em Ciências Biológicas - PPGCB, São Gabriel, Rio Grande do Sul, RS, Brazil.; 4 Universidade Federal do Paraná, Laboratório de Citogenética e Genética da Conservação Animal, Programa de Pós-graduação em Genética, Curitiba, PR, Brazil.; 5 Universidade Federal do Pará, Instituto de Ciências Exatas e Naturais, Belém, PA, Brazil.; 6 Instituto Evandro Chagas, Laboratório de Cultura de Tecidos e Citogenética - SAMAM, Ananindeua, PA, Brazil

**Keywords:** Charadrii, karyotype, Avian genome, comparative mapping

## Abstract

The order Charadriiformes comprises three major clades: Lari and Scolopaci as sister group to Charadrii. Until now, only three Charadriiformes species have been studied by chromosome painting: *Larus argentatus* (Lari), *Burhinus oedicnemus* and *Vanellus chilensis* (Charadrii). Hence, there is a lack of information concerning the third clade, Scolapaci. Based on this, and to gain a better understanding of karyotype evolution in the order Charadriiformes, we applied conventional and molecular cytogenetic approaches in a species belonging to clade Scolopaci - the wattled jacana (*Jacana jacana*) - using *Gallus gallus* and *Zenaida auriculata* chromosome-specific probes. Cross-species evaluation of *J*. *jacana* chromosomes shows extensive genomic reshuffling within macrochromosomes during evolution, with multiple fission and fusion events, although the diploid number remains at high level (2n=82). Interestingly, this species does not have the GGA7-8 fusion, which was found in two representatives of Charadrii clade, reinforcing the idea that this fusion may be exclusive to the Charadrii clade. In addition, it is shown that the chromosome evolution in Charadriiformes is complex and resulted in species with typical and atypical karyotypes. The karyotypic features of Scolopaci are very different from those of Charadrii and Lari, indicating that after divergence, each suborder has undergone different chromosome rearrangements.

## Introduction

Charadriiformes comprises 19 families with approximately 370 species (Gill and Donsker, 2017) and is divided into 3 clades: Lari (gulls, auks and their allies, along with buttonquails), Scolopaci (sandpipers, jacanas and allies), and Charadrii (plovers, oystercatchers and allies) (Baker *et al.*, 2007). Species of this order have been the subject of numerous studies, addressing topics such as systematics, behavior, diseases and cytogenetics (Baker *et al.*, 2007; Nie *et al.*, 2009; Bahl *et al.*, 2013; Kretschmer *et al.*, 2015b; Jackson *et al.*, 2017). Cytogenetics has shown the occurrence of a wide range of diploid numbers, from 2n=42 to 98 in *B*. *oedicnemus* (Nie *et al.*, 2009) and *Gallinago gallinago* (Hammar, 1970), respectively. However, the exact nature of the chromosomal rearrangements that took place in the karyotype evolution of Charadriiformes remains unclear, since only three species have been studied by chromosome painting. These studies have revealed that the reduction of diploid number in *B*. *oedicnemus* (Charadrii) was largely due to multiple fusions involving microchromosomes (Nie *et al.*, 2009). In *Larus argentatus* (Lari) 2n=70, only fusions of macrochromosomes (GGA5-9) with microchromosomes were detected (Hansmann *et al.*, 2009). On the other hand, in *Vanellus chilensis* (Charadrii) 2n=78, the only fusion observed was between GGA8/GGA7, and no fissions were detected (Kretschmer *et al.*, 2015b).[Bibr B7]

Comparative chromosome painting has contributed to the reconstruction of the evolutionary chromosomal history of birds (Griffin *et al.*, 2007; Furo *et al.*, 2015; Kretschmer *et al.*, 2015a, 2018a,b). Since the first production of chromosome-specific probes for *Gallus gallus* (GGA; Galliformes) in 1999 (Griffin *et al.*, 1999), other species have been chosen for the same purpose, such as *Burhinus oedicnemus* (Charadriiformes) (Nie *et al.*, 2009), *Leucopternis albicollis* (Accipitriformes) (de Oliveira *et al.*, 2010), and more recently, *Zenaida auriculata* (ZAU) (Columbiformes) (Kretschmer *et al.*, 2018b). ZAU has the same organization of macrochromosomes as proposed for the avian putative ancestral karyotype (PAK) (Griffin *et al.*, 2007) and is similar to *Gallus gallus* (the only difference being the fusion of PAK4 with PAK10, forming GGA4p and GGA4q, respectively). Furthermore, ZAU probes have the advantage over GGA probes in cross-species hybridization as they tend to produce stronger hybridization signals than *Gallus gallus* probes in Neognathae (Kretschmer *et al.*, 2018b), probably because the divergence between ZAU and other Neognathae species is more recent than chicken.

The wattled jacana (*Jacana jacana*) belongs to clade Scolopaci, and its karyotype has not been reported yet. It is an interesting species with a polyandrous mating system, in which a single female defends a harem of up to four males by aggressively excluding other females from their territory; males provide nearly all parental care (Osborne *et al.*, 1977; Emlen and Wrege, 2004). The purpose of this study was to perform comparative chromosome painting using whole chromosome probes of *Gallus gallus* and *Zenaida auriculata* in order to test: i) if a species of the clade Scolopaci has a chromosomal organization similar or different to the species of the clade Charadrii or Lari, and ii) if the karyotype of the *Jacana jacana* is reorganized by interchromosomal rearrangements, despite maintaining a conserved chromosomal morphology and diploid number.

## Material and Methods

### Sampling

Two males and two females of the *Jacana jacana* were sampled from São Gabriel, Rio Grande do Sul/Brazil. The collection and analyses were developed in agreement with SISBIO 44173-1 and Comissão de Ética no Uso de Animais- CEUA 018/2014 authorization.

### Acquisition of mitotic cells

Mitotic cells were obtained from fibroblast culture according to Sasaki *et al.* (1968). Briefly, a small skin sample of each specimen was collected and incubated in 2 ml of collagenase type IV (0.5%) for one hour at 37 ºC. The resulting cell suspension was washed in 5 mL of Dulbecco’s Modified Eagle Medium (DMEM) and centrifuged for 10 min at 800 rpm. Afterwards, the supernatant was removed and the cells were resuspended in 5 ml of DMEM, supplemented with 10% fetal calf serum (FCS) and 1% Penicillin-Streptomycin (10,000 U/mL), and incubated at 37 ^o^C. To arrest cells in metaphase, cultures were treated with 0.016% colchicine for 1 hour. After hypotonic treatment in 0.075 M KCL (15 minutes), cells were fixed in methanol:glacial acetic acid (3:1) and dropped onto clean slides.

### Chromosomal analysis

To determine the 2n and chromosome morphology, chromosome preparations were stained with 5% Giemsa solution in 0.07 M phosphate buffer (pH 6.8), and 40 metaphase spreads were analyzed for each specimen. The chromosome measurements for macrochromosomes (14 pairs) and sex chromosomes Z and W were made using ImageJ software (Rueden *et al.*, 2017). The remaining chromosomes were not measured because of their very small size, being considered microchromosomes. Karyotypes were arranged according to chromosome size and centromere position. Heterochromatic regions and W chromosome were identified by CBG banding performed according to Sumner (1972).

### Fluorescence *in situ* hybridization (FISH)

FISH experiments were performed using whole chromosome probes of *Zenaida auriculata* (ZAU1-5, which are homologous to GGA1-3,4q and 5, respectively) (Kretschmer *et al.*, 2018b) and *G. gallus* (GGA4, 6-16). Chromosome probes GGA15 and GGA16 were present in the same pool (GGA R5). Both sets of probes were labeled with biotin–dUTP or digoxigenin–dUTP, and the hybridizations were performed according to de Oliveira *et al.* (2010). After 3 days of hybridization at 37 °C, biotin-labeled probes were visualized using a layer of Cy3-streptavidin and digoxigenin-labeled probes with sheep anti-digoxigenin FITC coupled antibodes. After detection, chromosomes were counterstained with DAPI and examined by fluorescence microscopy.

Although we used ZAU1-5 probes, all karyotype comparisons were made with their homology with *G*. *gallus*, since most efforts to map the genome of birds have concentrated on the chicken, including the homology to the putative ancestral avian karyotype.

## Results

### Karyotype description

**Figure 1:**
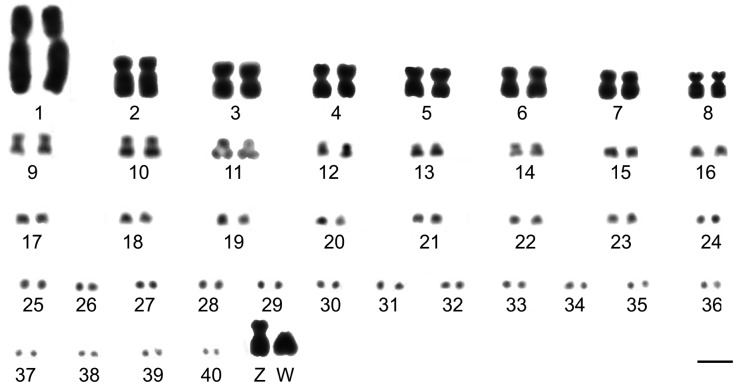
Conventional karyotype of a female individual of *Jacana jacana* with 2n=82. Bar = 5 μm.

*Jacana jacana* has 2n=82 chromosomes (Fig. 1). The karyotype comprises 14 pairs of biarmed macrochromosomes (including the sex chromosomes), the first pair being markedly larger than the other autosomes (Table 1). Pairs 2 to 8 are similar in size, as are the ninth to the eleventh. The latter (11th) can be distinguished from the others by a secondary constriction, containing the rDNA sequences. The Z chromosome is submetacentric and its size is equivalent to the autosomal pair 2. The W chromosome is acrocentric and it is smaller than Z.

**Table 1:** Measurements of 14 macrochromosomes and sex chromosomes ZW of *Jacana jacana*.

Chromosomes	p	q	p+q	IC	Morphology
1	357,00	558,00	915,00	0,39	Submetacentric
2	158,67	277,33	436,01	0,36	Submetacentric
3	180,00	217,33	397,33	0,45	Metacentric
4	162,67	193,34	356,01	0,46	Metacentric
5	144,00	186,00	330,00	0,44	Metacentric
6	142,67	184,00	326,67	0,44	Metacentric
7	124,01	185,33	309,34	0,40	Metacentric
8	110,00	173,00	283,00	0,39	Submetacentric
9	102,00	135,00	237,00	0,43	Metacentric
10	105,00	132,00	237,00	0,44	Metacentric
11	90,00	120,00	210,00	0,43	Metacentric
12	64,01	94,67	158,68	0,40	Metacentric
13	61,35	90,71	152,05	0,40	Metacentric
14	64,01	84,00	148,01	0,43	Metacentric
Z	149,33	237,33	386,67	0,39	Submetacentric
W	0,00	241,33	241,33	0,00	Acrocentric

p: short arm, q: long arm, p+q: total length, CI %: centromeric index=p/p+q × 100.

### CBG banding analysis

**Figure 2:**
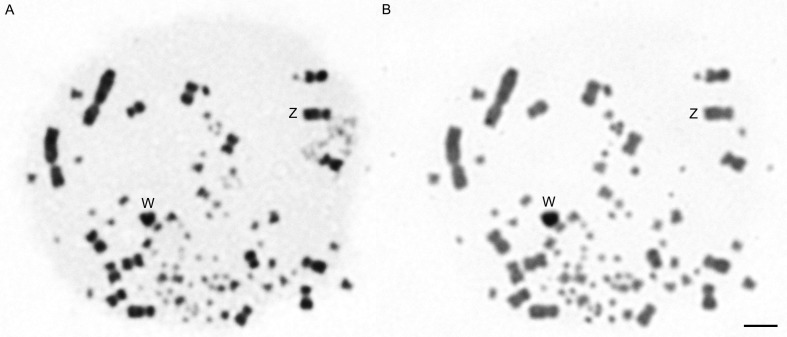
Metaphase of one female *Jacana jacana* in sequential Giemsa (A) and C-banding analysis (B) showing the Z and W sex chromosomes. Bar = 5 μm.

Sequential analysis of the same metaphase with Giemsa staining and C-banding confirms the identification of the W chromosome (Figure 2). The W chromosome is almost completely heterochromatic, whereas the autosomes do not show heterochromatic blocks.

### Comparative chromosome painting

**Figure 3:**
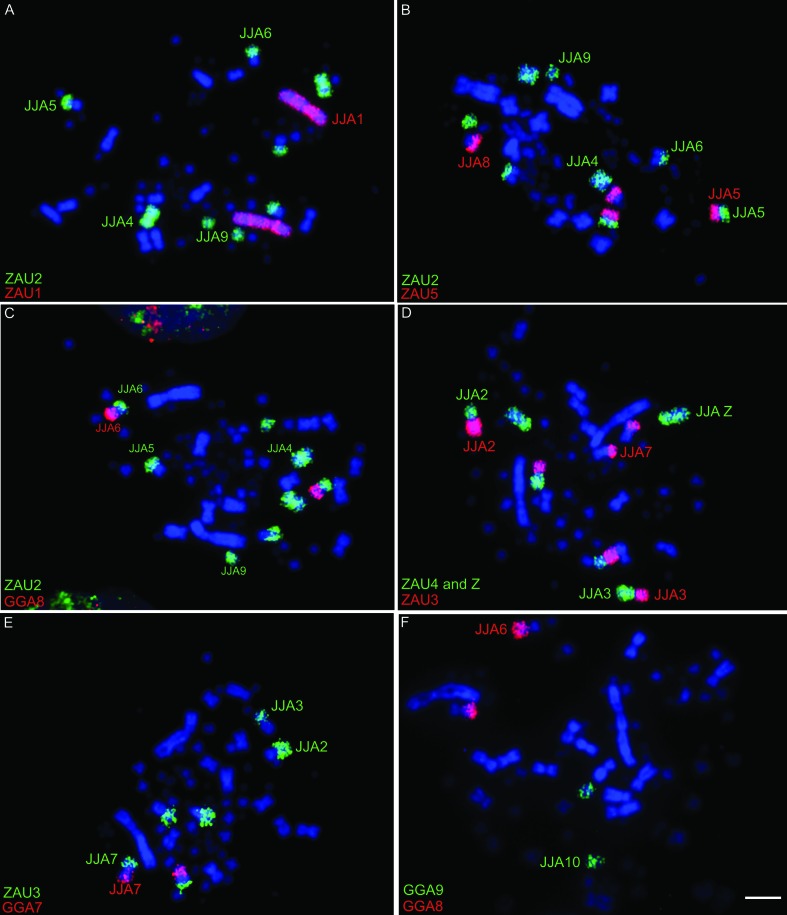
Chromosome painting with chicken and *Zenaida auriculata* (ZAU) probes to metaphase spreads of *Jacana jacana* (JJA) male. A) ZAU1 in red and ZAU2 in green; B) ZAU5 in red and ZAU2 in green; C) GGA8 in red and ZAU2 in green; D) ZAU3 in red and ZAU4 and Z in green; E) GGA7 in red and ZAU3 in green; and F) GGA8 in red and GGA9 in green. Biotin-CY3 (red) and digoxigenin-FITC (green). The chromosomes are stained with DAPI (blue). Bar = 5 μm.

**Figure 4:**
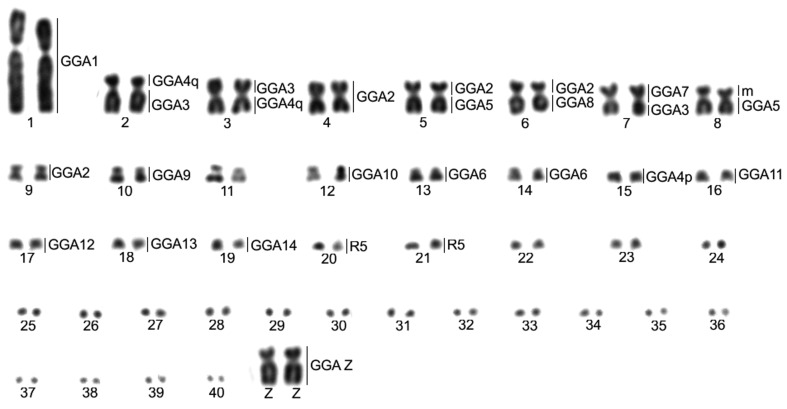
Karyotype of a male individual of *Jacana jacana*. Homology with *Gallus gallus* chromosomes (GGA) is indicated in bars on the right side of the corresponding chromosome. R5=GGA15 and 16, m= microchromosome. Bar = 5 μm.

Cross-species chromosome painting results show extensive genomic rearrangement of the ancestral chromosomes in *Jacana jacana* karyotype (JJA, Fig. 3 and 4). The fissions of GGA2 homologous chromosomes (4 pairs), GGA3 (3 pairs), GGA4 (3 pairs), GGA5 (2 pairs) and GGA6 (2 pairs) are evident. In addition, several chromosomal associations are observed in JJA: GGA2 (JJA4, JJA5p, JJA6p, JJA9); GGA3 (JJA2q, JJA3p, JJA7q); GGA4q (JJA2p, JJA3); GGA4p (JJA15); GGA5 (JJA5q, JJA8q); GGA6 (JJA13, JJA14), GGA7 (JJA7p), GGA8 (JJA6q). Chromosome painting also shows that the JJA karyotype has some fully conserved ancestral syntenies, corresponding to GGA 1 (JJA1), GGA9 (JJA10), GGA10 (JJA12), GGA11 (JJA16), GGA12 (JJA17), GGA13 (JJA18) and GGA14 (JJA19). Chromosomes GGA15 and GGA16 (GGA R5) are homologous to JJA20 and JJA21. Figure 4 shows the homology between the GGA1-16 and JJA.

## Discussion

Given that approximately 63% of birds have 2n=74-86 (Christidis, 1990), the chromosome organization of *Jacana jacana* resembles the typical avian karyotype at a first glimpse, with 2n=82 and comprising few macrochromosomes pairs (15 pairs, including Z and W chromosomes) and numerous microchromosomes (26 pairs). However, the chromosome morphology and size observed (Fig. 1) is different from other bird species, especially paleognathous birds, and even from species of Charadriiformes (Nishida-Umehara *et al.*, 2007; Hansmann *et al.*, 2009; Nie *et al.*, 2009; Kretschmer *et al.*, 2015b). The main difference is in the large number of medium biarmed chromosomes observed, which may be a result of chromosomal fusions or intrachromosomal rearrangements (centromere repositioning or inversions).

Chromosome painting indicates that *J*. *jacana* karyotype underwent extensive reorganization during its evolution from the PAK, mainly involving macrochromosomes. Hence, although pair 1 was entirely homologous to GGA1, pairs 2 to 6 (GGA2-6) show many fissions, sometimes in more than one segment. For instance, the ancestral chromosome 2 (GGA2) is split between 4 pairs and the ancestral 3 (GGA3) between three pairs, while ancestral pairs 4-6 (GGA4q-6) are split between two pairs each. However, despite the high frequency of chromosome fission in this species, the diploid number has been maintained close to the ancestral karyotype (2n=80) (Griffin *et al.*, 2007), due to several chromosomal fusions (Fig 1, 3 and Table 2). The associations between parts of ancestral chromosome 2 (GGA2) and 8 (GGA8) in JJA6 and between parts of ancestral 3 (GGA3) and 7 (GGA7) in JJA7 have not been described previously in bird species (reviewed in Kretschmer *et al.*, 2018a). These results highlight the value of chromosome painting in evolutionary cytogenetics, since a conserved diploid number does not always represent a conserved karyotype. It would be of interest to analyze if Charadriiformes species with high diploid numbers, but without fusions, have the same breakpoints as *J*. *jacana*, since some studies indicate that breakpoints recur in different avian lineages (Volker *et al.*, 2010; Warren *et al.*, 2010; Skinner and Griffin, 2012; Kretschmer *et al.*, 2018b).

**Table 2:** Chromosomal homologies among Charadriiformes species and *Gallus gallus* (GGA).

Chromosome	*B*. *oedicnemus*, 2n=42 (Nie *et al.*, 2009)	*V*. *chilensis*, 2n=78 (Kretschmer *et al.*, 2015b)	*J*. *jacana*, 2n=82 (Present study)	*L*. *argentatus*, 2n=70 (Hansmann *et al.*, 2009)
1	GGA1	GGA1	GGA1	GGA1
2	GGA2	GGA2	GGA3+GGA4q	GGA2
3	GGA3	GGA3	GGA3+GGA4q	GGA3
4	GGA4q	GGA7+GGA8	GGA2	GGA5+MIC
5	GGA7+8	GGA4q	GGA2+GGA5	GGA4q
6	GGA5	GGA5	GGA2+GGA8	GGA6+GGA9, R3 or R6
7	GGA9, R3+R6	GGA6	GGA7+GGA3	GGA7 or 8+GGA9, R3 or R6
8	GGA4p+R2	GGA9	GGA5+ 1MIC	GGA7 or 8+R4 or R1
9	GGA6+MIC	GGA10	GGA2	GGA4p or R2
10	R4+R1	-	GGA9	R7 or R2
11	R7+R2	-	-	GGA9, R3 or R6
12	R5+MIC	-	GGA10	R5 or MIC
13	R9+R6	-	GGA6	R5 or MIC
14	R5+MIC	-	GGA6	R7 or R6
15	R7	-	GGA4p	R6 or R9
16	R6	-	GGA11	R7 or R2
17	R9	-	GGA12	R5 or MIC
18	R9	-	GGA13	MIC
19	R9	-	GGA14	R7 or R6
20	R9	-	R5	-
21	-	-	R5	-

GGA R1, R3 and R4 each contain one microchromosome pair, GGA R2 and R5 each comprised two microchromosome pairs, GGAR6 included three microchromosome pairs and GGA R7 and R9 each contain multiple microchromosomes (Nie *et al.*, 2009). MIC= microchromosome.

Most of the microchromosomes are fully conserved in *J*. *jacana*. For instance, the use of the GGA1-16 chromosome probes showed the existence of only one gap in the JJA8 chromosome. Considering that 22 pairs of chicken microchromosomes (GGA17-38) were not studied, and 20 pairs of microchromosomes were left unhybridized by any of the probes used in *J*. *jacana*, it seem possible that at least two pairs of microchromosomes are involved in fusion events, one of them occurring in JJA8, which would correspond to GGA5 fused to a microchromosome.

This study on *J*. *jacana* is the first in which a representative of the clade Scolopaci has been analyzed by chromosome painting (Figure 3 and 4). In Charadriiformes, comparative chromosome mapping data are available for *B*. *oedicnemus* (Nie *et al.*, 2009) and *V*. *chilensis* (Kretschmer *et al.*, 2015b), both belonging to the Charadrii, and *L*. *argentatus*, belonging to the Lari (Baker *et al.*, 2007). A comparison of the chromosome painting data obtained to date shows that each species has a different genome organization (Table 2). Based on this, and considering the diploid number variation from 2n=42 in *B*. *oedicnemus* (Nie *et al.*, 2009) to 2n=98 in *Gallinago gallinago* (Hammar, 1970), we can assume that the order Charadriiformes underwent a unique chromosomal evolution. For instance, *V*. *chilensis* (2=78) can be considered a species with a more basal karyotype, since it has a relatively conserved diploid number, differing by only a fusion of the ancestral chromosomes 7 (GGA7) and 8 (GGA8) (Kretschmer *et al.*, 2015b). On the other hand, *L*. *argentatus* has a lower diploid number (2n=70) (Hansmann *et al.*, 2009), mainly due to the occurrence of fusions of macrochromosomes (GGA5-9) with microchromosomes. In contrast, *B*. *oedicnemus* shows a diploid number considered to be extremely low for the class Aves (2n=42). According to Nie *et al.* (2009), this divergence occurred through multiple fusions involving both microchromosome-microchromosome and microchromosome-macrochromosome fusion events, such as in GGA9, 2, 4p, 6 (Table 2) and macrochromosome-macrochromosome fusions without any identified chromosome fission (Nie *et al.*, 2009). In *J*. *jacana*, we can see a unique karyotype, resulting from fissions of the macrochromosomes, not previously reported for the order Charadriiformes, and fusions between the segments resulting from these fissions. Unfortunately, there are no chromosome-painting data for other species belonging to the Scolopaci, but it is possible that the associations observed in *J*. *jacana* are exclusive to the genus or even to the Scolopaci, since they were not observed in *B*. *oedicnemus*, *V*. *chilensis* (Charadrii), or *L*. *argentatus* (Lari). In addition, since *J*. *jacana* and *L*. *argentatus* do not have the fusion between ancestral chromosomes 7 and 8, the hypothesis that this fusion is an exclusive characteristic of the clade Charadrii is reinforced (Kretschmer *et al.*, 2015b).

**Figure 5:**
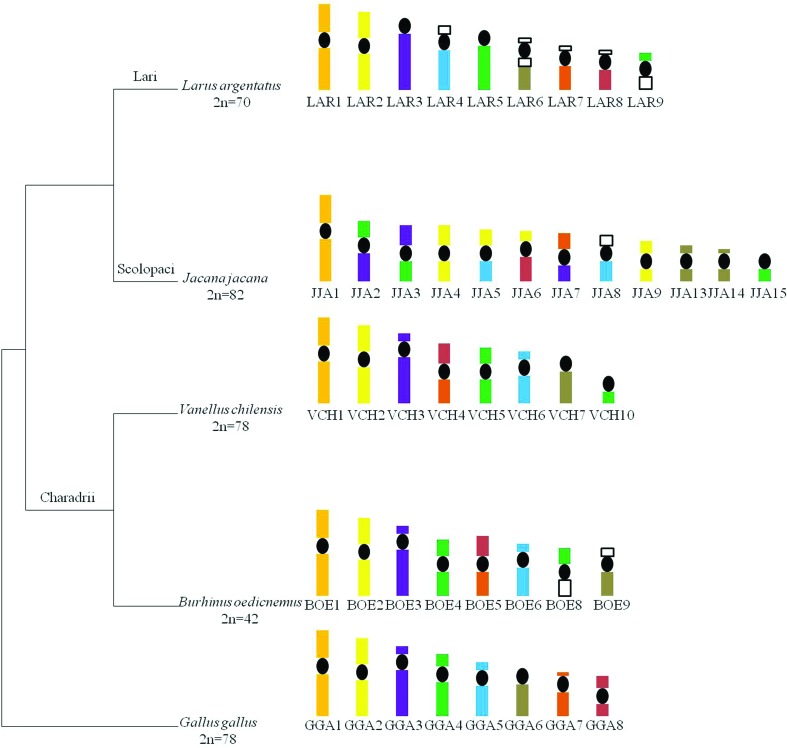
Phylogenetic relationships in the order Charadriiformes with schematic representation of the chromosome rearrangements on the right of the phylogeny. *Larus argentatus* data from Hansmann *et al.* (2009), *Vanellus chilensis* from Kretschmer *et al.* (2015b) and *Burhinus oedicnemus* from Nie *et al.* (2009). 2n = diploid number. Phylogenetic relationships follow Baker *et al.* (2007).

Considering that *Vanellus chilensis*, a member of clade Charadrii, which is placed in a basal position in the Charadriiformes phylogeny (Baker *et al.*, 2007), has a conserved karyotype (Kretschmer *et al.*, 2015b), we propose that the last common ancestor for the three suborders (Lari, Scolopaci and Charadrii) had a conserved karyotype, and after divergence, each suborder underwent different chromosomal rearrangements (Fig. 5 and Table 2). For instance, considering available results of chromosome painting, we can assume that genome organization in the suborder Lari has involved mainly fusion between microchromosomes and macrochromosomes, while in the suborder Scolopaci the main rearrangements were fissions and fusions between macrochromosomes. On the other hand, in the suborder Charadrii we see many fusions between microchromosomes, as observed in *B*. *oedicnemus*, or conserved karyotypes, such as observed in *V*. *chilensis*. Despite the fact that cytogenetic data in this group are still fragmental, they do not corroborate the proposal based on mitochondrial and nuclear sequences, which places the genus *Burhinus*, which has a highly diverged karyotype in a basal position, as sister group to the rest of the Charadrii, including *Vanellus* (Baker *et al.*, 2007) with a conserved karyotype.

In conclusion, despite the existence of chromosome-painting data for only a few species of the order Charadriiformes, an interesting pattern of genomic reorganization can be observed. Furthermore, the chromosome fusion between GGA7-8 in *B*. *oedicnemus* and *V*. *chilensis*, and the absence of this rearrangement in *L*. *argentatus* (Lari) and *J*. *jacana* (Scolopaci), supports the view that the clade Charadrii is monophyletic, agreeing with molecular data. As conventional staining data have shown, there is a marked diversity in chromosome number and morphology in species of this order. Therefore, an analysis of karyotypes of other Charadriiformes species by chromosome painting is essential to clarify the relationships between the species and to determine whether the biarmed elements found in different species correspond to homologous segments or involve different chromosomal rearrangements. In addition, *Leucopternis albicollis* and chicken bacterial artificial chromosome (BAC) probes may provide important information about the breakpoints and intrachromosomal rearrangements in Charadriiformes species.
